# Anti-Angiogenic Effect of Triptolide in Rheumatoid Arthritis by Targeting Angiogenic Cascade

**DOI:** 10.1371/journal.pone.0077513

**Published:** 2013-10-28

**Authors:** Xiangying Kong, Yanqiong Zhang, Chunfang Liu, Wei Guo, Xiangbin Li, Xiaohui Su, Hongye Wan, Yanqun Sun, Na Lin

**Affiliations:** Institute of Chinese Materia Medica, China Academy of Chinese Medical Sciences, Beijing, China; Rush University Medical Center, United States of America

## Abstract

Rheumatoid arthritis (RA) is characterized by a pre-vascular seriously inflammatory phase, followed by a vascular phase with high increase in vessel growth. Since angiogenesis has been considered as an essential event in perpetuating inflammatory and immune responses, as well as supporting pannus growth and development of RA, inhibition of angiogenesis has been proposed as a novel therapeutic strategy for RA. Triptolide, a diterpenoid triepoxide from Tripterygium wilfordii Hook F, has been extensively used in treatment of RA patients. It also acts as a small molecule inhibitor of tumor angiogenesis in several cancer types. However, it is unclear whether triptolide possesses an anti-angiogenic effect in RA. To address this problem, we constructed collagen-induced arthritis (CIA) model using DA rats by the injection of bovine type II collagen. Then, CIA rats were treated with triptolide (11–45 µg/kg/day) starting on the day 1 after first immunization. The arthritis scores (P<0.05) and the arthritis incidence (P<0.05) of inflamed joints were both significantly decreased in triptolide-treated CIA rats compared to vehicle CIA rats. More interestingly, doses of 11∼45 µg/kg triptolide could markedly reduce the capillaries, small, medium and large vessel density in synovial membrane tissues of inflamed joints (all P<0.05). Moreover, triptolide inhibited matrigel-induced cell adhesion of HFLS–RA and HUVEC. It also disrupted tube formation of HUVEC on matrigel and suppressed the VEGF-induced chemotactic migration of HFLS–RA and HUVEC, respectively. Furthermore, triptolide significantly reduced the expression of angiogenic activators including TNF-α, IL-17, VEGF, VEGFR, Ang-1, Ang-2 and Tie2, as well as suppressed the IL1-β-induced phosphorylated of ERK, p38 and JNK at protein levels. In conclusion, our data suggest for the first time that triptolide may possess anti-angiogenic effect in RA both in vivo and in vitro assay systems by downregulating the angiogenic activators and inhibiting the activation of mitogen-activated protein kinase downstream signal pathway.

## Introduction

Rheumatoid arthritis (RA) represents a chronic autoimmune disease featured as progressive destruction of cartilage and bone, predominance of pro-erosive mediators and presence of proliferative synovitis, which is characterized by angiogenesis and infiltration of inflammatory cells [1]. Angiogenesis is the growth of new blood vessels from existing ones and is an important aspect of new tissue development, growth, and tissue repair. It occurs since the early stage of various diseases including diabetes, cancer, and chronic inflammatory conditions [2]. In 1980, angiogenesis was originally identified as a feature of RA by detecting a low molecular weight angiogenic factor in synovial fluids from RA patients [3]. In recent years, many studies have demonstrated that angiogenesis is an essential event in perpetuating inflammatory and immune responses, as well as supporting pannus growth and development of RA [4]. Disrupting the formation of new blood vessel can prevent delivery of nutrients into the inflammatory site, and can also contribute to vessel regression and disease reversal. Therefore, inhibition of angiogenesis has been proposed as a novel therapeutic strategy for RA.

In the current treatments for RA, there are three kinds of therapeutic agents: disease modifying antirheumatic drugs (DMARDs), non-steroidal anti-inflammatory drugs (NSAIDs) and steroid and biological response modifiers. Regarding to their antiangiogenic effects, DMARDs such as methotrexate (MTX), sulphasalazine (SASP) and penicillamine have been shown to inhibit angiogenesis in experimental systems [5]; Bucillamine (BUC) and gold sodium thiomalate (GST) have been demonstrated to inhibit vascular endothelial growth factor (VEGF) production [6]; Infliximab, cyclosporin and endostatin also might be attributable to the downregulation of VEGF and RA-associated angiogenesis [7]. Although these drugs are all clinically used to relieve the severity of RA, slow this disease progression, and prevent the subsequent joint damage [8]–[9], their clinical use has been limited because of their adverse effects with a high frequency and high cost of treatment. Extracts of the Chinese herb Tripterygium Wilfordii Hook f. (TWHF), also known as “Lei Gong Teng”, are clinically used as one of the most common systemic treatments for (auto) immune disorders including RA, immune complex nephritis and systemic lupus erythematosus [10]–[11]. It has been extensively used for centuries in China because of its favorable cost-benefit ratio. In 2009, Goldbach-Mansky et al. [12], [13] performed clinical trails to validate the disease-modifying effects of extracts of TWHF on patients with RA. The treatment with this extract administered over 24 weeks may be both effective and safe in treating patients with active RA. The rapid improvement in function and pain, and the profound effect on inflammation may make it an attractive and affordable alternative to currently available agents. As an active compound of TWHF, triptolide is immunosuppressive, cartilage protective and anti-inflammatory in vivo and effective on both humans and animals inflicted by a range of inflammatory and autoimmune diseases, such as RA [14]–[16]. A large number of recent studies have found its mechanisms on the treatment of RA [17]–[22]. However, the role of triptolide in angiogenesis of RA is still unclear. Since triptolide has been indicated to act as a small molecule inhibitor of tumor angiogenesis in several cancer types [23]–[25] and anti-angiogenic therapy has been proven therapeutically effective in RA, we here intend to investigate whether triptolide possesses an anti-angiogenic effect in this disease.

## Materials and Methods

### 1. Ethics Statement

The study was approved by the Research Ethics Committee of Institute of Chinese Materia Medica, China Academy of Chinese Medical Sciences, Beijing, China. All animals were treated in accordance with the guidelines and regulations for the use and care of animals of the Center for Laboratory Animal Care, China Academy of Chinese Medical Sciences.

### 2. Animals

Eighty male DA/Bkl (DA) rats (8–12 week old) were obtained from Bantin & Kingman (Fremont, CA, USA). All rats were maintained in a room equipped with an air-filtering system, and the cages and water were sterilized.

### 3. Induction of CIA

CIA was induced as previously reported [9], [26]–[30]. Briefly, bovine type II collagen (Chondrex, Redmond, WA, USA) was dissolved in 0.01 M acetic acid overnight at 4°C. This was emulsified in an equal volume of incomplete Freund’s adjuvant (Chondrex, Redmond, WA, USA). The rats were immunized intradermally at the base of the tail with 0.1 ml of emulsion containing 100 µg of type II collagen. On day 7 after the primary immunization, the rats were boosted as the first time.

### 4. Treatment and Groups

Triptolide (purity >99.98%) was kindly provided by Professor Sui Lin (Fujian Institute of Medical Sciences, Fuzhou, China), and this is commercially available from Alexix Biochemicals (San Diego, CA, USA). It was dissolved in 0.05% DMSO (Sigma, St. Louis, MO, USA). The route of triptolide delivery was oral administration intragastrically using syringe feeding. Treatment was given daily for a period of 28 days from day 1 to day 28 of first immunization. The dosage range of triptolide treated in CIA rats (11–45 µg/kg) was 1.8-fold of that in CIA mice (8∼32 µg/kg) according to our previous study [20].

Eighty DA rats were divided into 5 groups with the equal number (n = 16): normal control group (Control), CIA model group (Vehicle), CIA rats treated with 11 µg/(kg·day) triptolide (Trip 11), 22 µg/(kg·day) triptolide (Trip 22), and 45 µg/(kg·day) triptolide (Trip 45).

### 5. Evaluation of CIA

Rats were observed once every 3–4 days after primary immunization. Arthritis severity was evaluated by arthritis scores which were performed by two independent, blinded observers. All 4 limbs of the rats were evaluated according to a visual assessment of inflammation or swelling and scored from 0 to 20 as the following scoring system [26]. For each limb, the ankle or wrist joint was scored 0–4 (0 = no swelling, 1 = minimal swelling, 2 = medium swelling, 3 = severe swelling, and 4 = severe swelling and non-weight bearing). The midfoot or mid-forepaw (extensor region) was similarly scored 0–4. The 3 distal joints of the 4 most lateral digits were scored 0–1 (0 = no swelling and 1 = swelling). This resulted in a maximum possible score of 20 per limb (e.g., maximum score of 4 each for the ankle and midfoot, and 12 for the 4 digits). The arthritis score was the total of the scores for all 4 limbs (maximum possible arthritis score 80). Arthritis incidence values are the number positive/total number in group.

### 6. Micro-CT Imaging

Three-dimensional reconstruction and images of the ankle joints were obtained by microfocal computed tomography (micro-CT, Explore Locus SP, GE, USA) at the day 28 of first immunization. Briefly, after the rats in different groups being sacrificed using ether anesthesia, the left hind paws were removed and fixed in 4% paraformaldehyde for 24 h. The samples were scanned by micro-CT. And the constructed three-dimensional image were obtained.

### 7. Histology and Histologic Scoring

Rats were sacrificed by cervical dislocation on day 28 of first immunization. Both hind limbs including the paws, ankles, and knees, were dissected, fixed immediately for 24 h in 4% paraformaldehyde, decalcified in 10% EDTA for up to 2 month at 4°C, and embedded in paraffin. Tissue sections (4 µm) were mounted on common slides for staining with hematoxylin and eosin (H&E). All sections were randomized and evaluated by two trained observers who were blinded to the treatment groups and the arthritis severity of each mouse. Minor differences between observers were resolved by mutual agreement. The synovial vascularity (angiogenesis) was scored as following : the number of vessels was counted in five high-power magnification fields of synovial tissue from joints and the mean used for analyses [31].

### 8. Histochemical and Immunohistochemical Analysis of Synovial Vascular Density in Inflamed Joints

In order to measure vessel density in synovial membrane tissues of inflamed joints, the polyclonal antibody (Abcam, Cambridge, MA, USA) recognizing the CD31 panendothelial antigen (platelet endothelial cell adhesion molecule) PECAM-1 was used for microvessel and single endothelial cell staining on 4 µm thick paraffin embedded sections of knee joints. With similar method of previous study [32], vessel density was measured by dividing the number of vessel-like structures in the synovial membrane tissue by the area of the synovial membrane tissue. Vessel-like structures were quantified according to size and included capillaries (small PECAM-1+ structures, 1–2 cells in size with a visible lumen), small vessels (<10 µm diameter), medium vessels (10∼50 µm diameter), and large vessels (>50 µm diameter). Because PECAM-1 can be expressed by some leukocyte populations associated with inflammation, single cells with positive PECAM-1 expression but without a defined lumen were not included in vascular measurements. The levels of erythrocyte-containing vessels in synovial membrane tissue were also determined on H&E-stained paraffin sections of joints, and confirmed results obtained by CD31/PECAM-1 immunohistochemical analysis.

### 9. Cell Culture

Human fibroblast-like synoviocytes of rheumatoid arthritis (HFLS-RA) were obtained from Cell Applications INC (USA). HFLS-RA from differernt 3 RA patients were used in our experiment (the Lot No. is 2030, 2259, 2884 respectively, Cell Applications INC, USA). The cells were cultured in sterile synoviocyte growth medium (Cell Applications, USA) supplemented with 100 U/mL 1 penicillin, 80 U/mL 1 streptomycin, 2 mM Gln-glutamine, 10% fetal bovine serum (FBS), and were maintained at 37°C in a humidified 5% CO_2_ incubator. HFLS–RA were used at passage numbers 4 to 8 in this study.

Human umbilical vein endothelial cells (HUVECs) were obtained from ScienCell INC (USA). The cells were cultured in sterile endothelial growth medium (ScienCell, USA) supplemented with 5% FBS, 100 U/mL 1 penicillin and 80 U/mL 1 streptomycin, and were maintained at 37°C in a humidified 5% CO_2_ incubator. HUVECs were used at passage numbers 4 to 6 in this study.

### 10. Cell Viability Assay

HFLS–RA (5×10^4^ cells/mL) were seeded in 96-well plates and starved in serum free sterile synoviocyte growth medium (supplemented with 100 U/mL 1 penicillin, 80 U/mL 1 streptomycin, 2 mM Gln-glutamine) for 24 h. Cells were then incubated with or without IL-1β (10 ng/mL, Peprotech INC,USA ) plus different concentrations of triptolide (1, 10 and 50 ng/mL) for 24 h. HUVEC (3×10^4^ cells/mL) were seeded in 96-well plates and starved in serum free sterile endothelial growth medium supplemented with 100 U/mL 1 penicillin and 80 U/mL streptomycin for 24 h. Cells were then incubated with or without VEGF_165_ (20 ng/mL, Peprotech INC,USA ) plus different concentrations of triptolide (1, 10 and 50 ng/mL) for 24 h. Cell viability was determined by 3-(4,5-dimethyl-2-thiazolyl)-2,5-diphenyl-2H-tetrazolium bromide (MTS) method using CellTiter 96® AQueous One Solution Cell Proliferation Assay (Promega, USA) according to the manufacturer’s instructions. The experiments were carried out 3 times in triplicate measurements.

### 11. Cell Chemotactic Migration Assay

The chemotactic migration of HFLS–RA and HUVEC were both detected using a Transwell chamber with polycarbonate filters (diameter 6.5 mm; pore size 8 µm) according to the previous studies [33]–[35]. For HFLS–RA, VEGF_165_ (20 ng/mL) prepared in synoviocyte growth medium was placed in the lower wells; In the upper wells, cells (at a final concentration of 5×10^4^ cells/mL) were suspended in serum free sterile synoviocyte growth medium containing triptolide (1, 10 and 50 ng/mL). For HUVEC, 500 µL ECM (containing 5% fetal bovine serum) and VEGF_165_ (20 ng/mL) prepared in sterile endothelial growth medium was placed in the lower wells; In the upper wells, cells (at a final concentration of 1×10^4^ cells/mL) were suspended in serum free sterile endothelial growth medium containing triptolide (1, 10 and 50 ng/mL). The chamber was incubated at 37°C for 4 h. The cells were fixed and stained with hematoxylin and eosin (H&E). Nonmigrating cells on the filter’s upper surface were removed using a cotton swab. Chemotaxis was quantified by counting the cells that migrated to the lower side of the filter using optical microscopy (magnification×200). Four random fields were counted for each assay. All experiments were done in triplicate. Mean ± SEM was calculated from independent experiments.

### 12. Cell Adhesive Assay

HFLS-RA (5×10^4^ cells/mL) and HUVEC (5×10^4^ cells/mL) were seeded in fibronectin (FN, 20 mg/mL) or bovine serum albumin (BSA, 10 g/L, used as negative control) coated 96-well plates and respectively incubated in sterile synoviocyte growth medium (supplemented with 100 U/mL 1 penicillin, 80 U/mL 1 streptomycin, 2 mM Gln-glutamine and 10%FBS) and sterile endothelial growth medium (supplemented with 100 U/mL penicillin, 80 U/mL streptomycin and 5% FBS) for 24 h. Cells were then incubated without or with interleukin (IL)1-β (10 ng/mL, Peprotech INC, USA ) plus with different concentrations of triptolide (1, 10 and 50 ng/mL) for 24 h. After treatment, cells were washed twice with PBS, then 200 µL of sterile medium and 10% (v/v) MTS reagent was added to the cells. All absorbances at 490 nm were measured with a microplate reader. Results were expressed as cell adhesiveness. All experiments were done in triplicate. Mean ± SE was calculated from independent experiments.

### 13. Tube Formation Assay

In order to examine the inhibitory effect of triptolide on HUVEC tube formation, the tube formation assay was performed as described previously [35]–[37]. Matrigel (10 mg/mL) was plated in 96-well culture plates and allowed to polymerise at 37°C in 5% CO_2_ humidified for 30 mins. HUVECs were trypsinised, and resuspended in sterile medium (supplemented with 5% FBS, 100 U/mL penicillin and 80 U/mL streptomycin). HUVECs (2×10^4^ cells/mL) were added to each chamber, followed by addition of various concentrations of triptolide (1, 10 and 50 ng/mL) with or without VEGF_165_ (50 ng/mL), then incubated for 24 h at 37°C in 5% CO_2_. After incubation, the capillary-like tube formation of each well in the culture plates was photographed using phase contrast microscopy. Tube formation were quantitated by counting the number of branch points. All experiments were done in triplicate. Mean normalized protein expression ± SEM was calculated from independent experiments.

### 14. Enzyme-linked Immunosorbant Assay

Sera were obtained from the rats on day 28 of first immunization. The expression levels of tumor necrosis factor (TNF)-α, IL-1β, VEGF in sera, TNF-α, IL-17, VEGF, Angiopoietin (Ang)-1and Ang-2 in supernatants of HFLS–RA cells, VEGFR and Ang-2 receptor (Tie2) in HUVEC cells [with different treatments: Control–normal cultured cells; Vehicle–IL-1β induced cells; Trip groups–cells treated with various concentrations of triptolide (1, 10 and 50 ng/mL)] were detected by ELISA assay (R&D, USA) according to the manufacturer’s protocol and absorbance was measured at 450 nm. All experiments were done in triplicate. Mean protein expression ± SEM was calculated from independent experiments.

### 15. Western Blot Analysis

The Western blot protocol and semiquantitative analysis were carried out following the protocol of our previous study [38]. The following antibodies were used: p-ERK antibody (rabbit polyclonal antibody, dilution 1∶100, Cell Signaling Technology Inc. USA), p-p38 antibody (rabbit polyclonal antibody, dilution 1∶200, Cell Signaling Technology Inc. USA), p-JNK antibody (rabbit polyclonal antibody, dilution 1∶100, Cell Signaling Technology Inc. USA) and GAPDH antibody (internal control, rabbit polyclonal antibody, dilution 1∶2000, Santa Cruz Biotechnology Inc. USA). All experiments were done in triplicate. Mean normalized protein expression ± SEM was calculated from independent experiments.

### 16. Statistical Analysis

The software of SPSS version 11.0 for Windows (SPSS Inc, IL, USA) was used for statistical analysis. Continuous variables were expressed as mean ± SEM. Arthritis incidence was analyzed by a chi-square test, and pathological score was analyzed by non-parametric Kruskal-Wallis test. Other data were performed using ANOVA followed by a post hoc test or Student’s t-test. Differences were considered statistically significant when *P* was less than 0.05.

## Results

### 1. Triptolide Decreases Severity of Arthritis in CIA Rats

**Figure 1:**
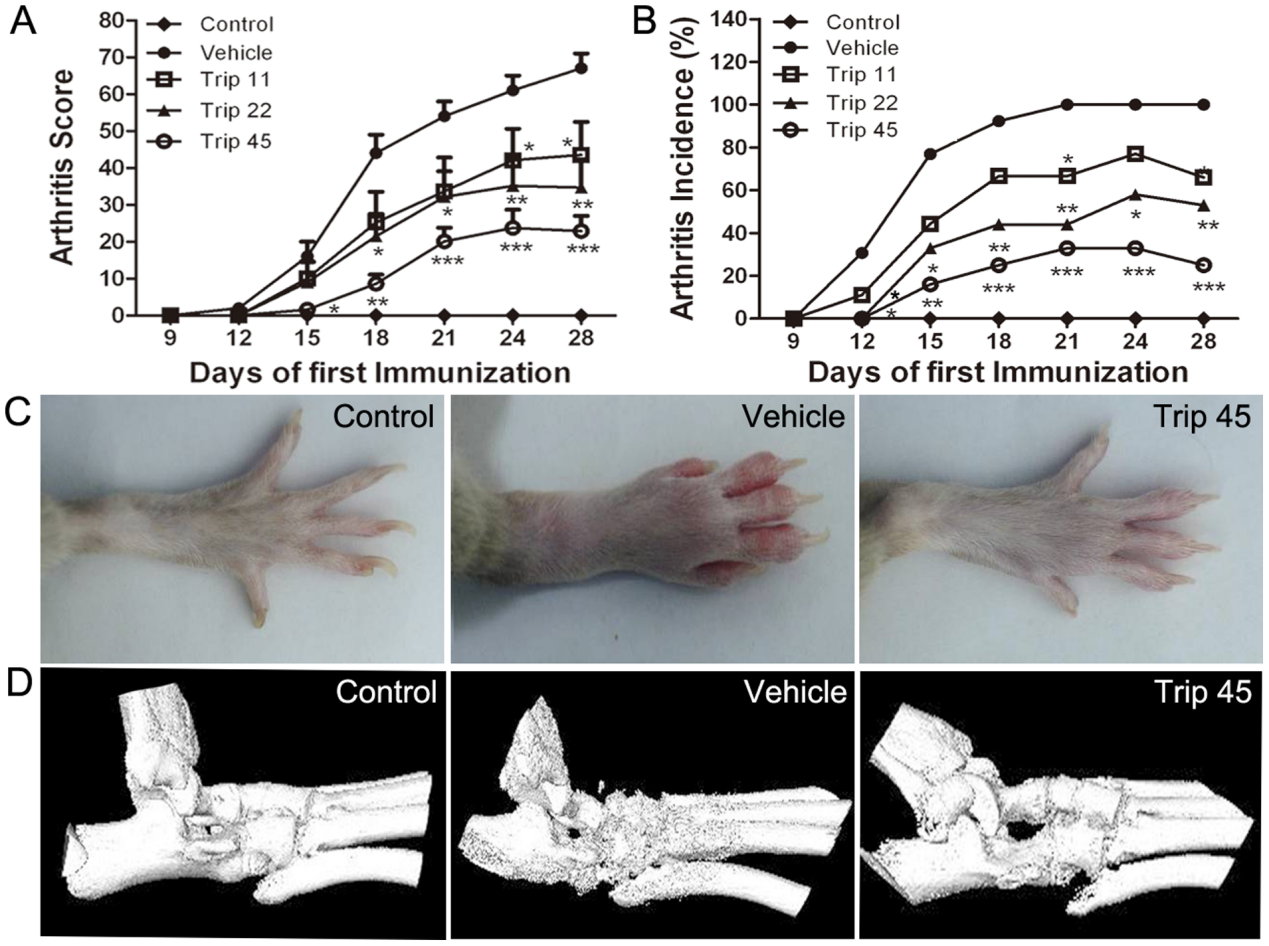
Triptolide decreases severity of arthritis in collagen-induced arthritis (CIA) rats. Rats were orally administered triptolide (Trip, 11, 22, and 45 µg/kg, respectively), or vehicle for 28 days from the day of first immunization. At the end of the experiment, the arthritis score and arthritis incidence were evaluated, and micro-CT scan was operated. (A) Doses of 11∼45 µg/kg triptolide significantly decreased the mean arthritis score in a dose-dependent manner compared with vehicle-treated rats; (B) Doses of 11∼45 µg/kg triptolide significantly decreased the arthritis incidence in a dose-dependent manner compared with vehicle-treated rats; (C) macroscopic evidence of arthritis such as erythema or swelling was markedly observed in untreated CIA rats, while doses of 45 µg/kg triptolide significantly attenuated arthritis severity in CIA rats; (D) the three-dimensionally reconstructed images of knee joints showed that dose of 45 µg/kg triptolide markedly reduced the extent of joint destruction compared with vehicle-treated rats. Data are represented as the mean ± SEM (n = 16). *P<0.05, **P<0.01, and ***P<0.001, comparison with the vehicle group.

To investigate the effect of triptolide on arthritis, the CIA model in DA rats was used. Oral administration of triptolide once a day started from day 1 to day 28 of first immunization. As shown in Figure 1A, triptolide dose-dependently interfered with increasing arthritis scores in CIA rats (all *P*<0.05). Consistent with the arthritis scoring, the assessment of arthritis incidence also showed triptolide to be highly effective (all *P*<0.05, Figure 1B); the incidence in the groups receiving triptolide (22–45 µg/kg) were markedly reduced from day 12 after first immunization. Additionally, macroscopic evidence of arthritis such as erythema or swelling and the evidence of joint destruction by Micro-CT scan were markedly observed in vehicle-treated CIA rats, while triptolide significantly attenuated arthritis severity in CIA rats (Figure 1C and 1D).

### 2. Triptolide Inhibits Angiogenesis in CIA Rats

**Figure 2:**
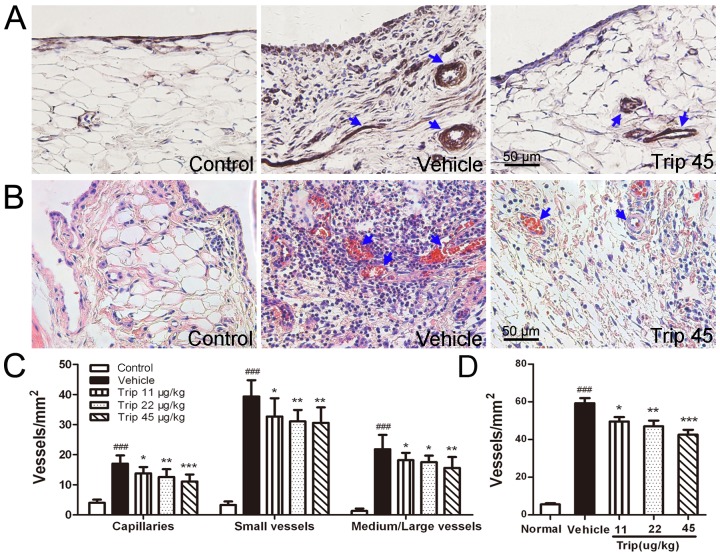
Vessel density in synovial membrane tissues of inflamed joints in collagen-induced arthritis (CIA) rats. (A), Platelet endothelial cell adhesion molecule 1 (CD31) immunohistochemical staining photomicrographs of synovial membrane tissues from knee joints of normal, CIA model and CIA rats treated with 45 µg/kg triptolide (Trip), respectively. (B), Hematoxylin and eosin staining photomicrographs of synovial membrane tissues from knee joints of normal, vehicle and 45 µg/kg triptolide-treated CIA rats, respectively. (C), Doses of 11∼45 µg/kg triptolide significantly decreased the capillaries, small, medium and large vessel density (CD31 immunohistochemistry) in synovial membrane tissues of inflamed joints in CIA rats. (D), Doses of 11∼45 µg/kg triptolide significantly decreased the vessel density (assessed on hematoxylin and eosin-stained paraffin sections) in synovial membrane tissues of knee joints in CIA rats. Data are represented as means ± SE (n = 16). ^###^ *P*<0.001, comparison with the control group. *P<0.05, **P<0.01, and ***P<0.001, comparison with the vehicle group.

Compared with vehicle-treated CIA rats, doses of 11∼45 µg/kg triptolide markedly reduced the capillaries, small, medium and large vessel density in synovial membrane tissues of inflamed joints in triptolide-treated CIA rats by immunohistochemical analysis (all *P*<0.05, Figure 2A and 2C). Findings were similar by histological evaluation (all *P*<0.05, Figure 2B and 2D). These results suggested that triptolide has a potent anti-angiogenic activity in vivo.

### 3. Triptolide Inhibits Chemotactic Migration of VEGF-induced HFLS–RA and HUVEC

**Figure 3:**
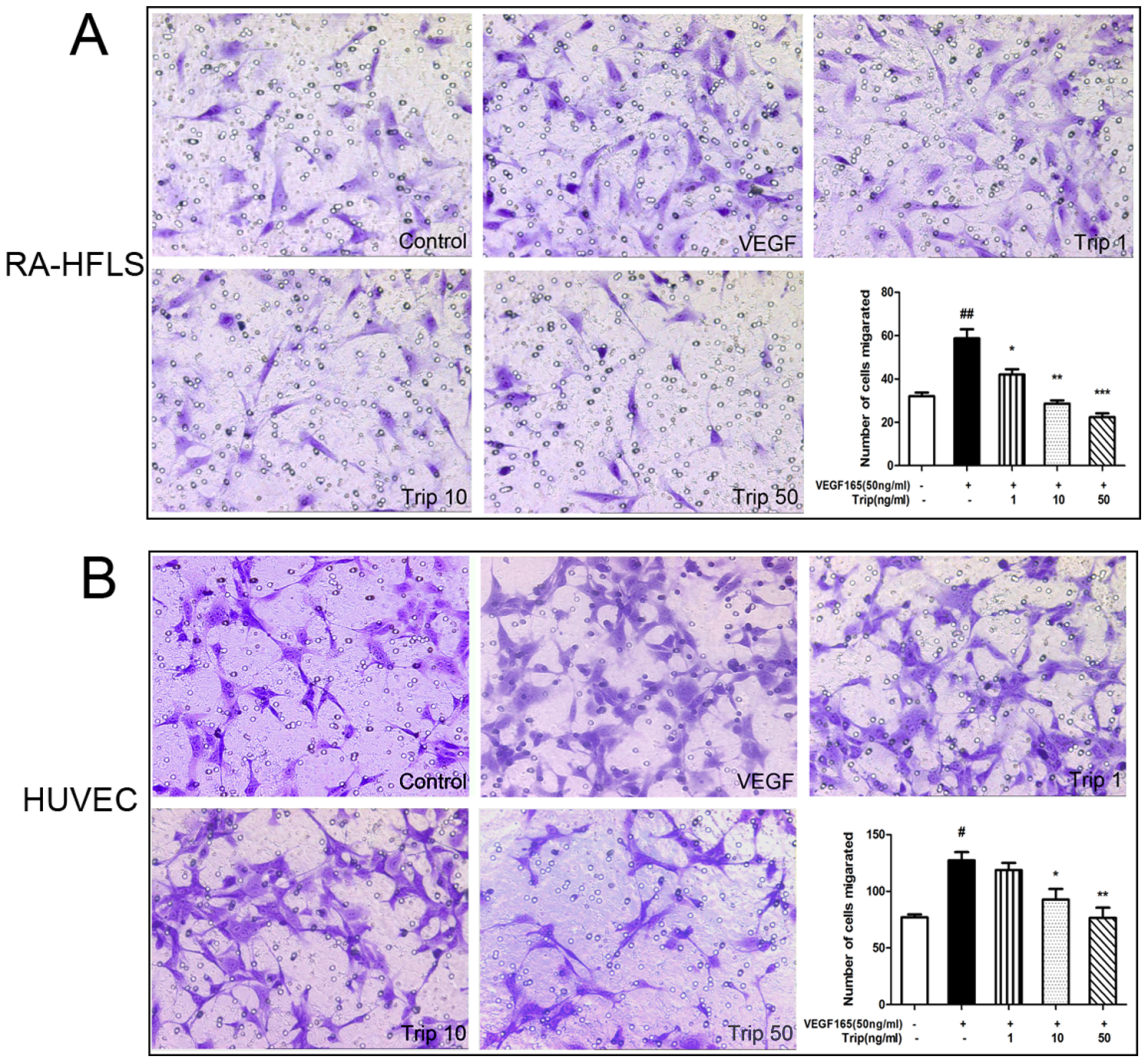
Inhibitory effect of triptolide on VEGF induced chemotatic migration of HFLS–RA (A) and HUVEC (B) using the Transwell culture insert. Cells were placed in transwells and allowed to migrate for 4(VEGF) with or without triptolide [Control, Vehicle and triptolide-treated (Trip) groups]. Migrated HFLS–RA and HUVECs were respectively fixed, stained and counted in eight random fields (magnification x40). All experiments were done in triplicate. Mean ± SE was calculated from independent experiments. ^#^ *P*<0.05 and ^##^ *P*<0.01, comparison with the control group. *P<0.05 and **P<0.01, comparison with the vehicle group.

Inhibitory effects of triptolide on the chemotatic migration of VEGF-induced HFLS–RA and HUVEC were demonstrated using the Transwell culture insert. As shown in Figure 3, the cell migration of HFLS–RA and HUVEC toward the chemo-attracting agent, VFGF, were both suppressed by triptolide with a dose-dependent manner (for HFLS–RA, Trip groups vs. Vehicle: *P*<0.05 or *P*<0.01 or *P*<0.001; Vehicle vs. Con: *P*<0.01; for HUVEC, Trip groups vs. Vehicle: *P*<0.05 or *P*<0.01; Vehicle vs. Con: *P*<0.05; Figure 3).

### 4. Triptolide Inhibits Cell Adhesion of HFLS–RA and HUVEC

**Figure 4:**
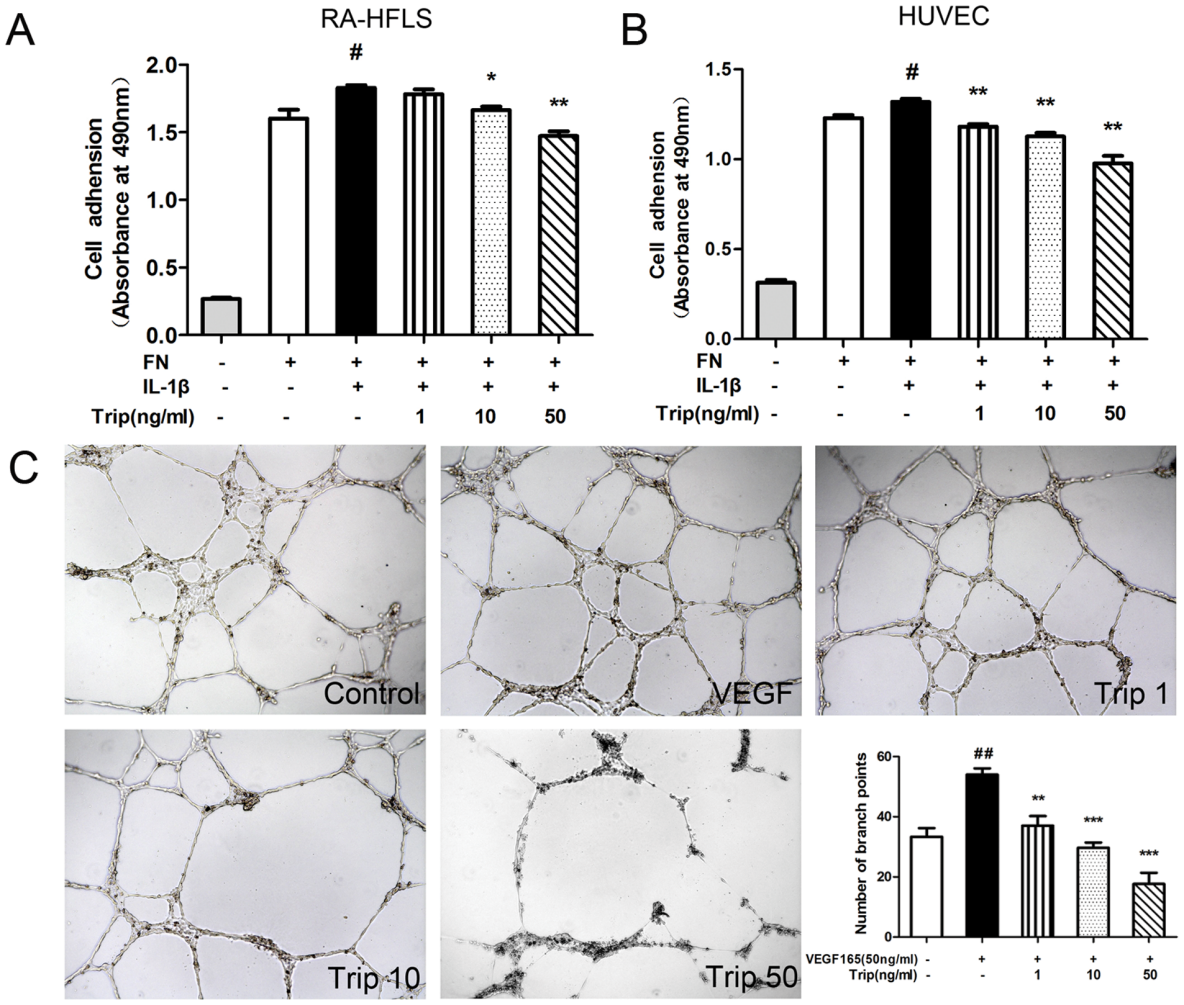
Inhibitory effects of triptolide on cell adhesiveness of HFLS–RA (A) and HUVEC (B) using the adhesive assay, and on tube formation of HUVEC (C). The cells were divided into six groups: negative control (cells were seeded in BSA coated 96-well plates), blank control (cells were seeded in FN coated 96-well plates), vehicle group (cells were seeded in FN coated 96-well plates with the presence of IL-1β), and three Trip groups (cells were seeded in FN coated 96-well plates with the presence of IL-1β and treated with 1, 10 and 50 ng/mL of triptolide, respectively). Compared with the vehicle group, triptolide at a concentration ranging from 1 to 50 ng/mL significantly suppressed the cell adhesiveness of HFLS–RA (P<0.01, A) and HUVEC (P<0.0, B) in a dose-dependent manner. (C) HUVEC were plated on the matrigel coated 96-well culture plates (Control), plated on the matrigel coated 96-well culture plates with the presence of VEGF (Vehicle), seeded in matrigel coated 96-well plates with the presence of VEGF and treated with 1, 10 and 50 ng/mL of triptolide, respectively(Trip 1, Trip 10 and Trip 50). Quantitation of the anti-angiogenic activities of triptolide on tube formation by counting the number of branch points, magnification x40. All experiments were done in triplicate. Mean ± SE was calculated from independent experiments. ^#^ *P*<0.05 and ^##^ *P*<0.01, comparison with the control group. *P<0.05, **P<0.01, and ***P<0.001, comparison with the vehicle group.

Inhibitory effects of triptolide on cell adhesiveness of HFLS–RA and HUVEC were determined by the adhesive assay. The cells were divided into six groups: negative control (cells were seeded in BSA coated 96-well plates), blank control (cells were seeded in FN coated 96-well plates), vehicle group (cells were seeded in FN coated 96-well plates with the presence of IL-1β), and three Trip groups (cells were seeded in FN coated 96-well plates with the presence of IL-1β and treated with 1, 10 and 50 ng/mL of triptolide, respectively). As shown in Figure 4, triptolide at a concentration ranging from 1 to 50 ng/mL significantly suppressed the cell adhesiveness of HFLS–RA (*P*<0.05 or *P*<0.01, Figure 4A) and HUVEC (*P*<0.01, Figure 4B) with a dose-dependent manner.

### 5. Triptolide Inhibits Tube Formation of HUVEC

The anti-angiogenic activities of triptolide on tube formation of HUVEC that mimics the angiogenic process were detected by the tube formation assay. Following plated onto matrigel, HUVECs quickly attached to the matrix and morphologically differentiated to a capillary-like network. As shown in the vehicle group, VEGF could promote the formation of capillary/tube-like networks of HUVEC. Compared with the vehicle group, triptolide-treated HUVEC formed relatively incomplete and narrow tube-like structures (Figure 4C). In addition, the anti-angiogenic activities of triptolide on tube formation were quantified by counting the number of branch points. As the results, triptolide at a concentration ranging from 1 to 50 ng/mL significantly reduced the extent of tubular formation of HUVEC with a dose-dependent manner (*P*<0.05 or *P*<0.01 or *P*<0.001, Figure 4C).

Nextly, we examined whether the above suppressive effect of triptolide was due to its cytotoxicity, since triptolide has been reported to induce apoptotic death of T lymphocytes [39]. When confluent HFLS-RA and HUVEC were treated with triptolide and/or IL-1β for 24 h, the cytotoxicity was monitored by MTS assay. Our results showed that triptolide did not exert any cytotoxic effects on HFLS-RA and HUVEC under the experimental conditions used in the present study (data not shown), which is consistent with our previous study [21], suggesting that triptolide might specifically suppress chemotactic migration, cell adhesion, and tube formation of HFLS–RA and HUVECs.

### 6. Triptolide Reduces the Levels of Various Angiogenic Activators

**Figure 5:**
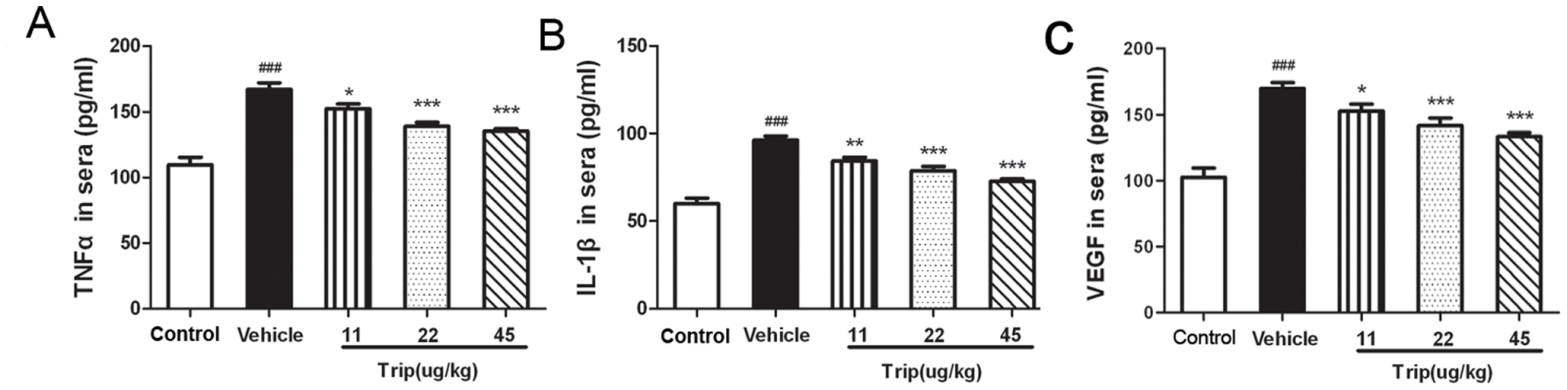
Triptolide reduces the expression levels of tumor necrosis factor (TNF)-α (A), Interleukin (IL)-1β (B) and VEGF (C) in sera of CIA rats. Rats were orally administered triptolide (Trip, 11, 22, and 45 µg/kg, respectively), or vehicle for 28 days from the day of first immunization. At the end of the experiment, sera were obtained from the rats and tested for TNF-α), IL-1β and VEGF by ELISA. All experiments were done in triplicate. ^##^ *P*<0.01 and ^###^ *P*<0.001, comparison with the control group. *P<0.05, **P<0.01, and ***P<0.001, comparison with the vehicle group.

**Figure 6:**
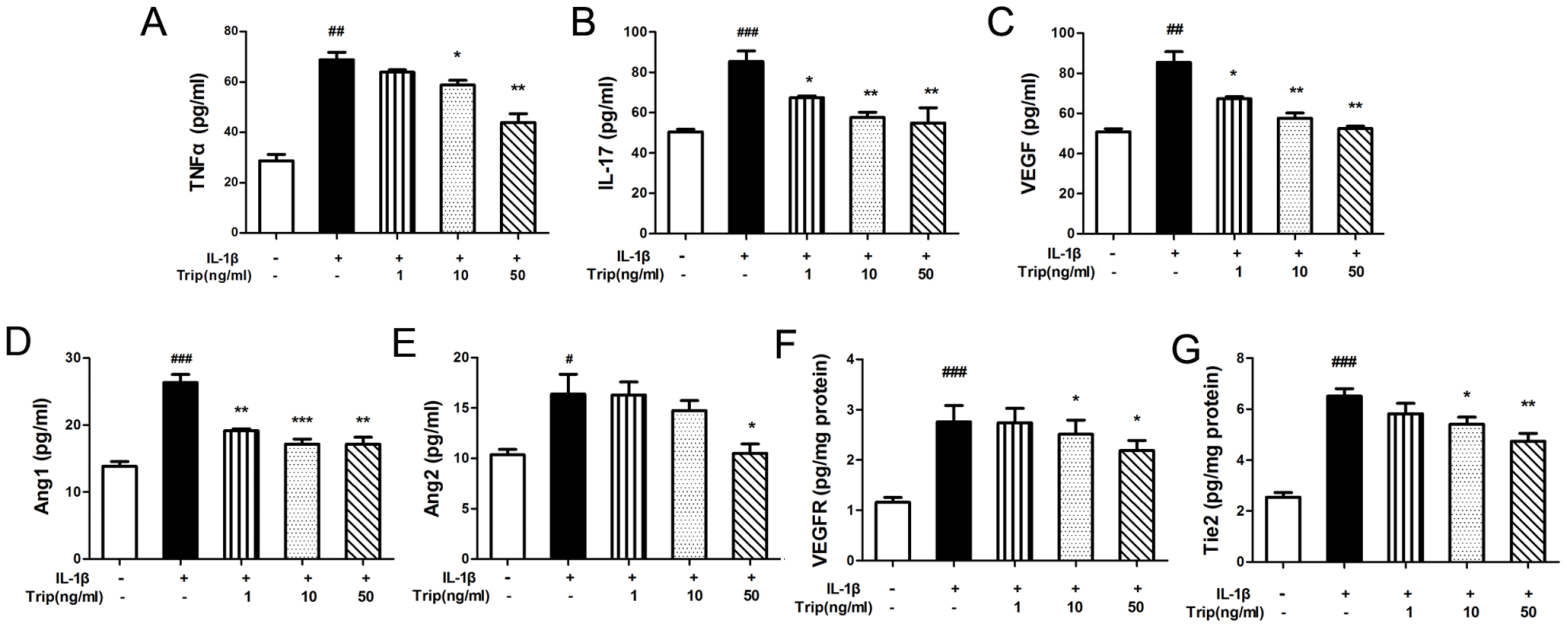
Triptolide reduces the expression levels of tumor necrosis factor (TNF)-α (A), Interleukin (IL)-1β (B), VEGF (C), Angiopoietin (Ang)-1 (D), Ang-2 (E) in supernatants of IL-1β-stimulated HFLS–RA cells, VEGF receptor (VEGFR, F) and Tie2 (G) in HUVEC. The cells were divided into five groups:Control–normal cultured cells; Vehicle–IL-1β induced cells; Trip groups–cells treated with various concentrations of triptolide (1, 10 and 50 ng/mL)] were detected by ELISA assay. All experiments were done in triplicate. Mean ± SE was calculated from independent experiments. ^#^ *P*<0.05, ^##^ *P*<0.01, ^###^ *P*<0.001, comparison with the control group. *P<0.05, **P<0.01, and ***P<0.001, comparison with the vehicle group.

In order to investigate the mechanisms by which triptolide suppressed the angiogenesis in RA, we detected the expression levels of angiogenic activators including TNF-α, IL-1β, VEGF in sera of rats, and TNF-α, IL-17, VEGF, VEGF receptor (VEGFR), Ang-1, Ang-2 and Tie2 in IL-1β-induced HFLS–RA/HUVEC by ELISA assay. Triptolide strongly reduced TNF-α (Figure 5A), IL-1β (Figure 5B), VEGF (Figure 5C) in sera of rats, and IL-1β-induced TNF-α (Figure 6A), IL-17 (Figure 6B), VEGF (Figure 6C) and Ang-2 (Figure 6E) secretion by HFLS–RA cells with a dose-dependent manner. Regarding to Ang-1, its expression levels in triptolide-treatment groups were all significantly lower than those in IL-1β group (*P*<0.05, Figure 6D). In addition, we also found that the expression levels of VEGFR (Figure 6F) and Ang-2 receptor (Tie2, Figure 6G) in HUVEC cells treated with 10∼50 ng/mL triptolide were significantly lower than those in the vehicle group.

### 7. Triptolide Suppresses IL1-β-induced Activation of MAPK Downstream Pathway

**Figure 7:**
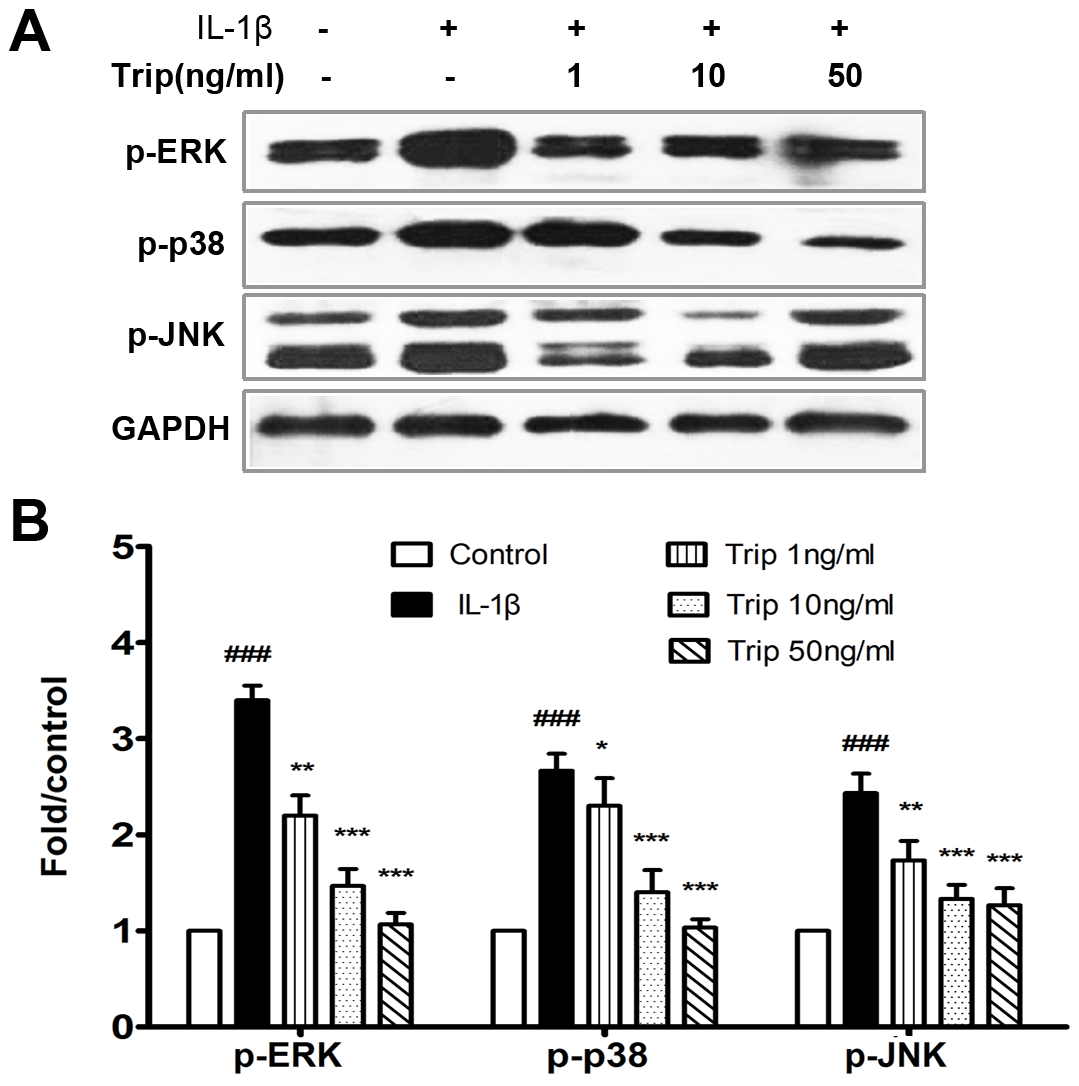
Triptolide reduced IL-1β-activated p-ERK, p-p38 and p-JNK in HFLS–RA cells detected by western blot analysis. Confluent HFLS-RA at passage 4–8 were incubated with various concentrations of triptolide (1, 10 and 50 ng/mL, respectively) for 24 h, followed by the addition of IL1-β (10 ng/mL). After 15 min incubation, cells were analyzed for detection of p-ERK, p-p38 and p-JNK by western blot analysis. All experiments were done in triplicate. Mean ± SE was calculated from independent experiments. ^###^ *P*<0.001, comparison with the control group. *P<0.05, **P<0.01, and ***P<0.001, comparison with the vehicle group.

Data aforementioned indicated that triptolide may inhibit various cellular functions including chemotactic migration, cell adhesion, and tube formation of IL1-β or VEGF-stimulated HFLS–RA and HUVECs. In order to investigate whether triptolide could modulate the activation of IL1-β induced signal pathways, we further detected the expression levels of p-ERK, p-p38 and p-JNK in IL1-β-induced HFLS–RA cells. As shown in Figure 7, triptolide markedly diminished the activation of IL1-β-induced ERK (*P*<0.01 or *P*<0.001), p38 (*P*<0.05 or *P*<0.001) and JNK (*P*<0.01 or *P*<0.001), which are associated with survival, migration and adhesion of HFLS–RA cells.

## Discussion

Triptolide, as a well-known immunosuppressive and anti-inflammatory agent, has been extensively used in the treatment of RA [14]–[19]. It is of great importance to understand its mode of actions and potential drug targets in order to effectively use triptolide for clinical therapy of RA. We have previously demonstrated that triptolide may exert chondroprotective and anti-inflammatory effects in RA by the direct suppression of the production of MMPs and the simultaneous up-regulation of TIMPs production in the joints of CIA mice [20]–[21], and also may prevent the bone destruction and inhibit osteoclast formation by regulating RANKL/RANK/OPG signal pathway [22]. In order to further clarify the mechanisms of triptolide acting on RA, we here discovered and demonstrated the anti-angiogenic effects of triptolide in RA. The main findings of our study are as following four points: (1) Triptolide attenuates RA partially by preventing the formation of new blood vessels in vitro and in vivo; (2) Triptolide may inhibit IL1-β-induced cell adhesion of HFLS–RA and HUVEC, as well as disrupt tube formation of HUVEC on Matrigel and suppress the chemotactic migration of VEGF-induced HFLS–RA and HUVEC, without any cytotoxic effects on these cells; (3) Triptolide may suppress IL-1β-induced upregulation of angiogenic activators including TNF-α, IL-17, VEGF, VEGFR, Ang-1, Ang-2 and Tie2 in sera of CIA rats and/or in HFLS–RA; (4) Triptolide may inhibit IL-1β-mediated activation of MAPK downstream signal pathway by reducing the expression of p-ERK, p-p38 and p-JNK. These findings suggest that triptolide may exert a novel anti-angiogenic activity on RA and its action may be partially caused by inhibiting various angiogenic activators and MAPK downstream signal pathway.

Angiogenesis as a critical component of disease progression in RA involves in the formation and maintenances the infiltration of synovial membrane. Following the improved understanding of the molecular mechanisms supporting the pathogenesis of RA, accumulating novel targets for the therapy of this disease have been discovered. Among them, RA-associated angiogenesis is one such novel target for disease modulation. In recent studies, triptolide has been confirmed to suppress angiogenesis of various human cancers including anaplastic thyroid carcinoma [23]–[24], lung cancer [25] and hematologic malignancies [39]. It is envisaged that triptolide may exert certain anti-angiogenic effects in the treatment of RA. At first, we validated that triptolide attenuated the severity of arthritis in CIA rats by reducing the mean arthritis score as well as arthritis incidence with a dose-dependent manner, which is consistent with the data of our previous studies [20]. Secondly, our results indicated that synovial vessel density in arthritic joints of CIA rats treated with triptolide was significantly decreased when compared with control joints. Besides, similar effect of triptolide was also found in the in vitro assay system. Three different functional assays were performed to confirm the anti-angiogenic activities of triptolide. The inhibitory effect of triptolide on the chemotactic migration of VEGF-induced HFLS–RA and HUVEC was found in the chemotaxis assay. The tube formation of HUVEC on the Matrigel depends on migration and morphological differentiation which mimics the process of angiogenesis. The incomplete and relatively narrow network-like structures formed by the triptolide-treated HUVEC indicated that such in vitro behaviors of HUVEC would be altered by triptolide. As the process of tube formation is highly relied on cell-cell adhesion [40], we also found that the cell adhesiveness of HFLS–RA and HUVEC on the Matrigel could be significantly reduced in the presence of triptolide. Under physiological condition, endothelial cells are often in a quiescent state. However, they are the direct and absolutely necessary executors in angiogenic cascade. After being activated by angiogenic factors, such as IL1-β, TNF-α and VEGF, endothelial cells are recruited to proliferate, migrate, form tube-like structure and eventually form blood vessels [41]. Since these activities of endothelial cells are essential for sustained angiogenesis, the inhibitory effects of triptolide on them clearly indicates its anti-angiogenic potentials.

We further explored the precise mechanisms involved in the anti-angiogenic activity of triptolide in RA. A great number of pro-angiogenic factors, including fibroblast growth factors, VEGF, Angs, epidermal growth factor, IL-1, IL-8, IL-17, TGF-α and TNF-α, govern angiogenesis in RA. These factors play important roles in the development of neovasculature by interacting with each other. The key signaling system that regulates proliferation and migration of endothelial cells forming the basis of any vessel are VEGF and their receptors. The VEGF-dependent signaling system is necessary for neoangiogenesis. In RA synovium, VEGF is produced by macrophages, vascular smooth muscle cells, synovial lining cells, fibroblasts surrounding microvessels, neutrophils of synovial fluid and peripheral blood mononuclear cells [42]. IL-17, as a proinflammatory cytokine that is implicated in the inflammation and destruction of the joint, has been demonstrated to increase the production of VEGF in RA [43]. Besides, the Tie/Ang cascade is another signaling system involved in regulation of complex interactions between endothelium and surrounding cells [44]. In this system, Angs play a crucial role in the control of vessel stabilization and regression. Despite structural similarity, Ang-1 and Ang-2 exhibit differently directed action on the Tie2-associated signaling cascade. Ang-2 is a competitive inhibitor of Ang-1. While Ang-1 stimulates Tie2 phosphorylation, interaction with Ang-2 does not result in activation of the receptor [45]. Ang-1 acts as stabilizer of new vessels elicited by VEGF, while Ang-2 destabilises these vessels, resulting in new vessel sprouts in the presence of VEGF, or to vessel regression in the absence of VEGF [46]. Moreover, TNF-α has also been indicated to induce the release of VEGF in RA, and the TNF-α blockade may disturb the balance of vessel growth and regression [47]. In the present study, our data showed that triptolide could suppress the levels of TNF-α, IL-1β, VEGF in sera of CIA rats and the IL-1β-induced upregulation of TNF-α, IL-17, VEGF, VEGFR, Ang-1, Ang-2 and Tie2 in HFLS–RA, suggesting the inhibitory effect of triptolide on the VEGF-mediated signal pathway. Given recent studies have demonstrated that triptolide may inhibit the production of TNF-α and IL-1β via interference with the transcriptional activation of NF-kB in the joints of CIA mice or rats [9], [20], the anti-angiogenic effect of triptolide might be related to the inhibition of NF-kB activation. In addition to these findings, we also observed that triptolide could inhibit the IL1-β-induced activation of MAPK downstream signal pathway, which is central to endothelial activation and has been used as an attractive target for the development of angiogenesis inhibitors [48].

In conclusion, our data suggest for the first time that triptolide may possess anti-angiogenic effect in RA both in vivo and in vitro assay systems by downregulating angiogenic activators and inhibiting the activation of MAPK downstream signal pathway. These findings provide a novel insight into the role of triptolide in RA pathogenesis and suggest that it might be an attractive and suitable therapeutic agent for treating this disease.
